# Glenoid wear and migration pattern of a humeral head resurfacing implant: a prospective study using radio stereometric analysis

**DOI:** 10.1016/j.jseint.2024.07.012

**Published:** 2024-08-05

**Authors:** Michael Axenhus, Magnus Ödquist, Hassan Abbaszadegan, Olof Sköldenberg, Björn Salomonsson

**Affiliations:** Department of Clinical Sciences at Danderyd Hospital, Karolinska Institutet, Stockholm, Sweden

**Keywords:** Arthroplasty, Glenoid wear, Gleno-humeral osteoarthritis, Humeral head resurfacing, Migration pattern, Radio stereometric analysis

## Abstract

**Background:**

The humeral head resurfacing arthroplasty (HHR) is normally used as a hemi shoulder arthroplasty and has been in use for the treatment of Gleno-Humeral osteoarthritis (OA) of the shoulder for more than 30 years. Some studies, however, shows that anatomical total shoulder arthroplasty provides better improvement in function than a HHR for patients with OA. Reasons for this may be a progressive glenoid wear (GW) or loosening of the HHR. We, therefore, wanted to investigate the migration pattern of the HHR and also GW by using radio stereometric analysis (RSA).

**Methods:**

21 patients (21 shoulders) with OA and a mean age of 64 years were enrolled in the study. They all received the Copeland humeral resurfacing head and were followed for 2 years with RSA. We evaluated the clinical outcome at 2 years with Western Ontario Osteoarthritis of the Shoulder (WOOS), EuroQol 5 dimension 3L and Constant Shoulder Score. In addition, we assessed data on WOOS and revisions until 5 years follow-up by using the local clinic data within the Swedish Shoulder Arthroplasty Register.

**Results:**

After an initial migration at two months the implants were stable in relation to the humerus with no statistically significant difference between the 2 months and the 2 years value (*P* = .23). The GW continued to increase during the study period with an initial migration of mean 2.3 mm and at 2 years 3.5 mm with a statistically difference between the 6 months and 2 years value (*P* = .046). The WOOS, EuroQol 5 dimension 3L and Constant Shoulder Score were all improved at 2 years compared to the preoperative values. We found a weak correlation between GW at 2 years and the WOOS score at 2 and 5 years, but these did not reach statistical significance. There were 4 revisions within 5 years after the primary operation, all due to pain.

**Conclusion:**

The marker-free RSA can be used in clinical studies for assessing migration in HHR implants and was also for the first time used to measure GW. The Copeland HHR seems to obtain a secure fixation in the humerus but shows continuous GW up to two years.

Gleno-humeral osteoarthritis (OA) of the shoulder causes pain and limited function and can be treated with shoulder arthroplasty.^17^^,^^27^ The humeral head resurfacing arthroplasty (HHR) is most often used as a hemi shoulder arthroplasty and has been in use with good results for the treatment of OA for more than 30 years.^9^^,^^12^^,^^20^ The advantage of HHR includes the possibility to restore normal anatomy with less surgical trauma and a possible future revision might be less technically challenging. There are, however, several studies that shows that anatomical total shoulder arthroplasty provides better improvement in function than HHR for patients with OA.^3^^,^^21^ A reason for this may be painful glenoid wear (GW) of the HHR. Radio stereometric analysis (RSA) is a reliable and well documented radiographic method for evaluation of fixation and migration of orthopedic implants. Early micromovements of an implant may predict future loosening.^10^

The aim of this study was primarily to evaluate the fixation of the Copeland HHR on the proximal humerus and to investigate the migration pattern and GW up to 2 years with RSA. To our knowledge no previous studies have assessed GW for hemi shoulder arthroplasty with RSA. As a secondary outcome we correlated the RSA results with the clinical outcome and revisions until 5 years after surgery.

## Materials and methods

### Study design and setting

This prospective cohort study was performed between 2009 and 2016 at Danderyd hospital, Stockholm, Sweden with patients recruited between 2009 and 2010. Danderyd hospital is a University teaching hospital with a catchment area of 700.000 inhabitants. The study was performed in accordance with the Helsinki declaration and the Ethics Committee of the Karolinska Institutet approved the study (No. 2008/1621-31/2). The results are presented in accordance with the Strengthening the Reporting of Observational Studies in Epidemiology criteria.^6^

### Inclusion and data collection

All patients referred to the orthopedic department at Danderyd Hospital for OA in the shoulder, were eligible for the study. We included patients aged 50-85 year old, with primary or secondary OA of the shoulder not responding to conservative treatment. We excluded severely ill patients not suited for surgery, those with severe destruction of the glenoid surface or cuff tear arthropathy, and patients not able to follow the study protocol.

All patients gave informed consent prior to any study-specific visits and were followed postoperatively by research nurses at regular follow-up visits. All data were collected in a patient specific case report form.

### Surgical technique

We used a standard deltopectoral approach. 1.0 mm Tantalum markers were inserted with a “bead-gun” and were placed in the proximal humerus, the coracoid and around the glenoid. All patients received the same implant, Copeland Humeral Resurfacing Head (Zimmer Biomet, Warsaw, IN, USA). Learning curve for the implants was not included in this study. Three surgeons performed all procedures (HA, MÖ, BS). Postoperatively the arm was placed in a double sling for 6 weeks and the patients were allowed to elevate the arm forward. All patients were instructed by an experienced physiotherapist postoperatively.

### Outcomes

**Figure 1:**
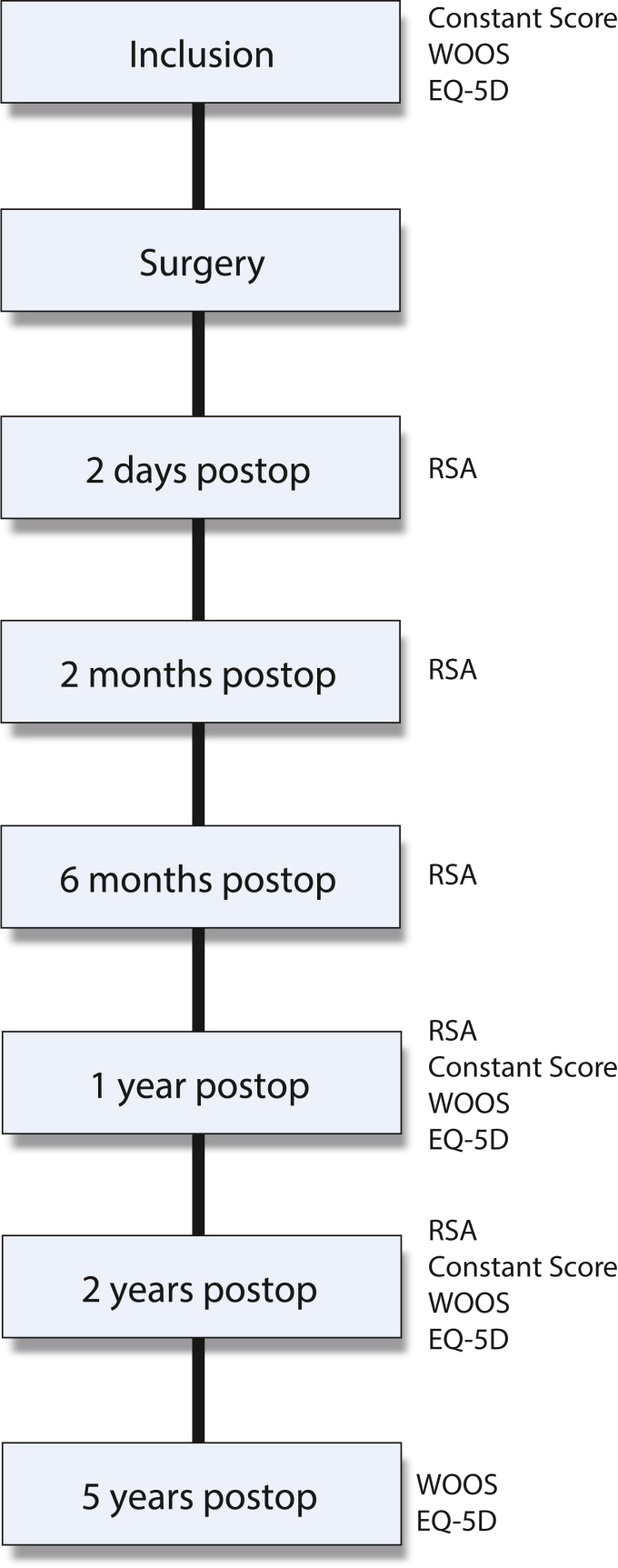
Study visits and data collection. *RSA*, Radio stereometric analysis; *WOOS*, Western Ontario Osteoarthritis of the Shoulder, *EQ-5D*, *EuroQol 5 dimension 3L*.

The main outcomes were the total migration (the combined three-dimensional (3D) vector of x-, y- and z-translation) of the HHR in relation to the proximal humerus and the glenoid, measured with RSA at 2 years. The secondary outcomes were functional outcome measured with Constant Shoulder Score (CSS) and patient-reported outcome measure scores where we used Western Ontario Osteoarthritis of the Shoulder (WOOS) and *EuroQol 5 dimension 3L* [EQ-5D]). We also correlated the total migration and GW to the 5 year WOOS and EQ-5D collected through the clinics access to the Swedish Shoulder Arthroplasty Registry (SSAR). In addition, we checked for revisions up till 5 years postoperatively (Fig. 1).

### Radio stereometric analysis

RSA is a high-precision method of assessing 3D micromotions from calibrated stereo radiographs and is used for evaluating new implants and we followed the published guidelines for RSA.^32^

For the RSA follow-ups we used digital calibrated radiographs, a uniplanar calibration cage (Uniplanar digital 43; RSA Biomedical AB, Umeå, Sweden) and analyzed all data using the UmRSA software (RSA Biomedical AB, Umeå, Sweden). The 1.0 tantalum markers implanted during surgery was used as reference for the measurements. The examinations were performed with the patient in a supine position and with the operated arm in a shoulder immobilizer.

Based on one of our earlier studies,^28^ marker-free RSA can be used for HHR implants. The method is not precise enough to measure rotations, thus only translations are used in this study as a proxy for the overall migration of the implants. The translations of the calculated center of gravity of the HHR in relation to either the proximal humerus segment or the glenoid segment was calculated at each follow-up visit and compared with the immediate postoperative measurements. For the GW, the 2 months RSA examination was used as a reference examination to exclude the distention of immediate postoperative intra-articular joint effusion.

At the 1 year follow-up we performed double examinations 10 min apart on all patients with complete repositioning of the X-ray tubes and the calibration cage. We calculated the precision as the 99% confidence interval (standard deviation [±SD] 2.7) of the difference between the examinations and found it to be between 0.14 and 0.33 mm for migration of the HHR in relation to the proximal humerus and 0.62 to 0.92 mm for GW (Table I). The mean error of rigid body fitting was used to evaluate the stability of the markers over time and, per recommendations from the RSA guidelines, we excluded examinations in which this value was >0.3 mm as this indicates migration of the markers. Precision measurements of RSA was done via double examinations fifteen minutes apart at the 1-year follow-up on all patients with complete repositioning of the X-ray tubes and the calibration cage. We used these measurements to calculate the precision as the 99% confidence limit (±SD 2.7) of the difference between the examinations.

**Table I:** Precision measurements of the RSA.

Precision (mm)	99% CL (±SD 2.7)
HHR vs. proximal humerus segment	
Medio-/lateral translation (x)	0.14
Superio-/inferior translation (y)	0.05
Antero-/posterior translation (z)	0.15
Total (x, y, z)	0.33
HHR vs. glenoid segment (glenoid wear)	
Medio-/lateral translation (x)	0.75
Superio-/inferior translation (y)	0.62
Antero-/posterior translation (z)	0.67
Total (x, y, z)	0.92

*RSA*, radio stereometric analysis; *HHR*, humeral head resurfacing arthroplasty; *CL*, confidence limit; *SD*, standard deviation.

Double examinations fifteen minutes apart were done at the 1-year follow-up on all patients with complete repositioning of the X-ray tubes and the calibration cage. We used these measurements to calculate the precision as the 99% confidence limit (±SD 2.7) of the difference between the examinations.

### Clinical evaluation and patient-reported outcome measures

WOOS, EQ-5D and CSS were collected before surgery and at the 1 and 2 years follow-up. WOOS and EQ-5D were also collected at 5 years through the SSAR.

The WOOS^13^ is a validated, patient-reported and disease-specific questionnaire for measurement of the outcome after shoulder arthroplasty. The result is usually combined to a single score representing the percentage of a healthy shoulder from 0% to 100%.

The CSS^4^ is an outcome measurement tool for assessing shoulder function.^11^ It assesses four aspects related to shoulder pathology; two subjective pain and activities of daily living which can give up to 35 points, and two objective, range of motion and strength which can give up to the objective 65 points. Thus, the resulting maximum total score is 100 points (best function). Pain and activities of daily living are answered by the patient, range of motion and strength requires a physical evaluation, and was performed by two experienced physiotherapists in this study.

The EQ-5D is a standardized instrument to measure the generic quality of life.^7^ It consists of 5 dimensions with questions which then can be combined to a single index ranking from −0.54 (worse than death when below zero) to 1 (best imaginable health state).

### Revisions

We double checked for any revisions up to 5 years postoperatively through our clinics access to the SSAR. Revisions were defined as removal, exchange, or addition of an implant component according to Nordic Arthroplasty Register Association.^22^

### Sample size and statistics

A power calculation with the assumption that with 90% power and a *P* value of <.05 using total migration of the implant as outcome, showed that 13 patients must be included to detect a translation of the HHR in relation to the humerus and to detect any medial migration into the glenoid. This calculation assumed a precision of the RSA method for total migration of 0.3 mm with an SD of 0.3 and was derived from a previous publication from our group. The RSA method is complicated with an expected technical loss of at least 20% of the examinations, and we, therefore, planned to include approximately 25 patients in the study. The Student’s t-test was used for comparative analysis, a *P* value of <.05 was considered significant.

## Results

### Participants and descriptive data

**Figure 2:**
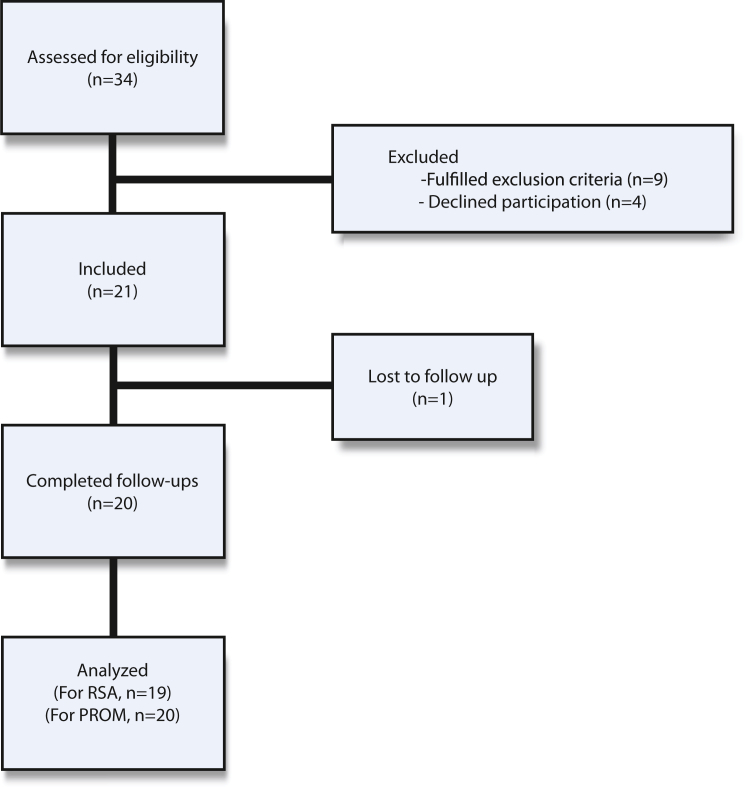
Flowchart of patients in the study.

We enrolled 21 patients (mean age 64, male:females, 11:10) in the study. (Fig. 2, Table II). There were no deaths during the study period.

**Table II:** Baseline characteristics of the patients.

	All	Women	Men
	(n = 21)	(n = 10)	(n = 11)
Age, years (mean, SD)	64 (±9)	69 (±7)	60 (±10)
Arthroplasty on the other side, yes	6	5	1
Side R/L	11/Oct	05/May	06/May
Preop EQ-5D (mean, SD)	0.440 (±0.327)	0.475 (±0.332)	0.406 (±0.335)
Preop constant (mean, SD)	29 (±9)	26 (±8)	31 (±10)
Preop WOOS% (mean, SD)	41% (±11)	40% (±11)	43% (±12)
Preop Copeland strl (median, range)	4 (3-8)	4 (3-4)	6 (4-8)

*EQ-5D*, *EuroQol 5 dimension 3L*; *SD*, standard deviation; *preop*, preoperative; *WOOS*, Western Ontario Osteoarthritis of the Shoulder.

#### Radio stereometric analysis

**Figure 3:**
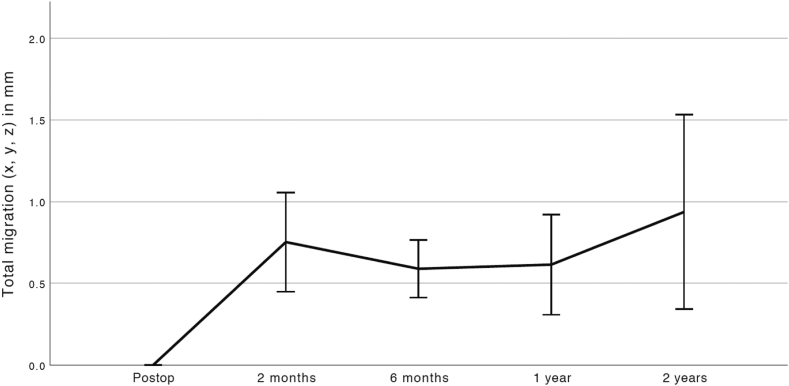
Line chart of the mean (and 95% CI) in total translation resurfacing head in relation to the humeral shaft. After the initial migration up to 2 months there was no statistically significant migration at subsequent follow-ups indicating firm fixation of the resurfacing head. *CI*, confidence interval.

After an initial migration at 2 months the implant was stable at 2 years in relation to the humerus with no statistically significant difference between the 2 months and 2 years value, *P* = .226 (Table III, Fig. 3). All but 2 implants showed a migration above the detection limit of RSA at 2 years and 3 implants had migrated above 1.0 mm with 1 implant showing a maximum migration of 4.7 mm. All data points are provided as supplementary material (Supplementary Table S1).

**Table III:** Main outcomes for the RSA analysis in mm.

Outcome (mm)	Mean	SD	95% lower CL	95% upper CL	Min	Max	n	*P* value
Total migration (x,y,z)								
2 mo	0.75	±0.50	0.43	1.07	0.34	2.10	12	
6 mo	0.61	±0.30	0.43	0.78	0.17	1.23	14	
1 y	0.60	±0.53	0.31	0.89	0.09	2.44	15	
2 y	0.94	±1.14	0.33	1.54	0.14	4.70	16	.226^a^
Glenoid wear (x,y,z)								
6 mo	2.32	±1.42	1.41	0.49	0.49	4.97	12	
1 y	2.95	±2.00	1.84	4.05	1.03	7.14	12	
2 y	3.45	±1.91	2.53	4.37	1.03	7.14	19	.046^a^

*CL*, confidence limit; *RSA*, radio stereometric analysis; *SD*, standard deviation.

*P* values are derived from the Student's t-test. a = compared to the 2 months value.

**Figure 4:**
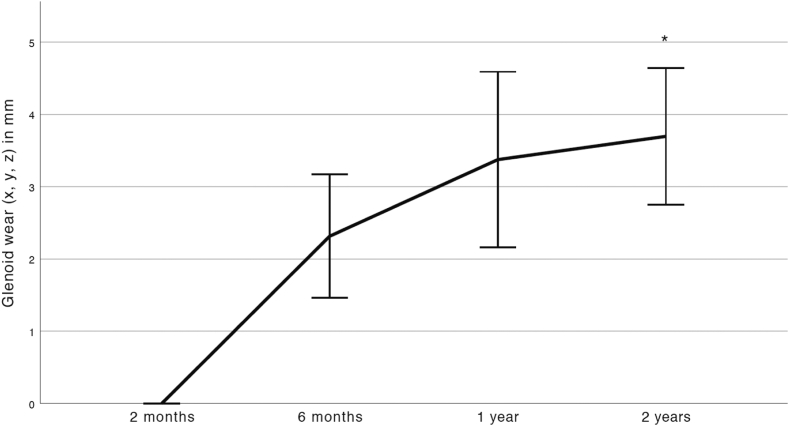
Line chart of the mean (and 95% CI) wear of the resurfacing head into the glenoid. The 2 months postoperative RSA examination is used as baseline. The asterisk (*∗*) indicates statistical significance compared to the 6 months value with the Student's t-test at *P* ≤ .05. *CI*, confidence interval; *RSA*, radio stereometric analysis.

The GW was continuous with an initial migration of mean (SD) 2.3 (±1.4) mm and then increasing to 3.5 (±1.9) mm at 2 years with a statistically significant difference between the 6 months and 2 years value, *P* = .046 (Table III, Fig. 4). All implants showed GW above the detection limit of RSA at 2 years with 7 out of 19 (37%) implants having GW of over 5 mm.

#### Clinical evaluation and patient-reported outcome measures

**Figure 5:**
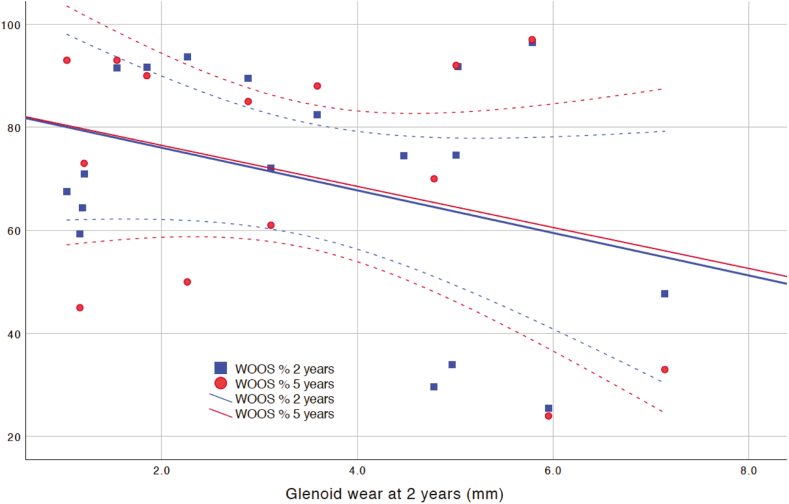
Scatterplot of GW at 2 years vs. the 2 and 5 years WOOS score with regression lines and mean 95% CI. We found a weak correlation between GW at 2 years and the WOOS score at 2 and 5 years, but these did not reach statistical significance (R^2^ = 0.125, *P* = .15 and (R^2^ = 0.125, *P* = .15 and R^2^ = 0.11, *P* = .17 at 2 and 5 years, respectively). *CI*, confidence interval; *WOOS*, Western Ontario Osteoarthritis of the Shoulder.

The CSS, WOOS, and EQ-5D all improved compared to the preoperative values (Table IV). We found a weak correlation between GW at 2 years and the WOOS score at 2 and 5 years, but these did not reach statistical significance (R^2^ = 0.125, *P* = .15 and R^2^ = 0.11, *P* = .17 at 2 and 5 years, respectively) (Fig. 5). There was no correlation between fixation of the implant in the humerus and any clinical outcome score.

**Table IV:** Clinical outcomes.

	Mean	Standard deviation	N	*P* value
Constant Score				
Preoperatively	29	±9	21	
1 y	50	±15	17	
2 y	52	±15	19	<.001
WOOS%				
Preoperatively	41%	±11	18	
1 y	72%	±23	19	
2 y	69%	±22	20	
5 y	70%	±23	16	.001
EQ-5D index				
Preoperatively	0.44	±0.33	20	
1 y	0.58	±0.28	15	
2 y	0.54	±0.34	17	
5 y	0.64	±0.31	17	.06

*EQ-5D*, *EuroQol 5 dimension 3L*; *WOOS*, Western Ontario Osteoarthritis of the Shoulder.

*P* values are derived from the Student’s t-test and compared to the preoperative value.

#### Revisions

There were 4/21 (19%) reoperations and 3 revisions within 5 years after the primary operation. One arthroscopic reoperation was due to frozen shoulder with stiffness. The 3 revisions were caused by painful glenoid erosion, and they were all revised to stemmed TSA.

## Discussion

### Radio stereometric analysis

The main findings of our study were that the implant fixation in the humerus can be considered as adequate, thus implant loosening was not a clinical problem in this study, and also that all patients developed GW. We have shown that with RSA it is possible to study implant migration in the shoulder using a model based algorithm, this has also been shown by Mechlenburg et al and Stilling et al.^14^^,^^30^ Our results show that RSA is a reliable method to measure GW in HHR, and in addition also the direction of the GW. The most common way to measure GW is by plain radiographs.^18^^,^^31^ A study by Mercer et al,^15^ presented a method where they used standardized anteroposterior and axillary radiographs and then manually measured the position of the humeral head center in relation to scapular reference coordinates. We believe that RSA is a more accurate and precise method, but more costly and time-consuming. Computed Tomography^5^ or multichannel computed tomography^16^ may also be used to measure GW and loosening after HHR. In a study by Parsons et al, they used a Microscribe 3-DX (GoMeasure3D, Amherst, VA, USA) digitizing device were used with a reported precision of 0.23 mm.^19^ We believe that RSA is a more accurate and precise method, but more costly and time-consuming. Furthermore, recent advances in RSA technology have enabled accurate methodology with limited radiation exposure.^26^

GW was detectable in all patients and in 37% (7/19) patients it was more than 5 mm. This is similar to what previous studies have found.^8^^,^^25^^,^^33^ The prevailing direction of the GW was posterior and superior, indicating that the RH may cause an asymmetrical GW over time which might influence both the clinical outcome and the surgical demands if revision becomes indicated.

In a retrospective study by Al-Hadithy they reviewed 53 Copeland HHR at a mean follow-up of 4.2 years and concluded that Copeland HHR can provide functional results similar to modular stemmed prostheses.^1^ Rasmussen et al showed a mean WOOS score of 67 one year after surgery.^23^ Our clinical results were similar to these two studies.

### Clinical outcome

We found a weak correlation between GW at 2 years and the WOOS score at 2 and 5 years, but it did not reach statistical significance. We believe that the number of patients in the study was not sufficient to prove or dismiss a possible correlation. The values for CSS and WOOS improved postoperatively and were stable until 2 years and for WOOS also at 5 years. That is in line with earlier publications.^12^^,^^23^ In a systematic review and meta-analysis from Bryant et al they concluded that in short-term follow-up of 2 years, total shoulder arthroplasty provides more consistent improvement in function than hemiarthroplasty for patients with primary OA of the shoulder.^3^

### Revisions

19% (4/21) of the patients were revised within 5 years after the primary operation due to painful GW. In a study by Rasmussen et al from the Danish Shoulder Arthroplasty Registry they found that 7.5% of HHR needed revision until 5 years after operation.^24^ Soudy et al, found 17% revisions after 56 months of which 9.5% where due to GW.^29^ A recent thesis written by ten Brinke described an incidence of 17% (4/24) when it came to loosening of the implant in HHR.^2^

The strength of our study is that with the precision of the used RSA method we were able to show that GW is a reality for all patients operated with a hemi HHR. And it is also a strength that we for the first time show that it is possible to use marker-free RSA to study both implant fixation and GW in a clinical study of shoulder HHR. The weaknesses of this study of implant fixation and GW is that we only had 21 patients which was not enough to show a significant correlation between GW, and clinical outcomes such as pain. The study is not randomized, lacks a control group, and has a short follow-up time. Moreover, we cannot make accurate measurements of rotations by the implant with marker-free RSA.

## Conclusion

The marker-free RSA can be used in clinical studies for assessing migration in HHR implants and was also for the first time used to measure GW. We conclude that the Copeland HHR, after an initial migration, obtains a secure fixation in the humerus. But the Copeland HHR shows continuous GW, and GW is known to be a clinical problem which may lead to revision to a TSA. Therefore, careful consideration must be taken when deciding which type of implant to choose in a patient with primary OA.

## Acknowledgment

The authors acknowledge their research nurses, Helené Sjöö and Paula Kelly-Peterson, for all the work keeping track of the patients in this study. They also acknowledge their physiotherapists, Gunilla and Gunilla, who performed all the physical examinations for the Constant Score.

## Disclaimers:

Funding: This study was supported by grant from 10.13039/501100004047Karolinska Institutet and by the regional agreement on medical training and clinical research in the Greater Stockholm area (ALF). Zimmer-Biomet partially funded the RSA-examinations but had no further input on the study.

Conflicts of interest: The authors, their immediate families, and any research foundation with which they are affiliated have not received any financial payments or other benefits from any commercial entity related to the subject of this article.
